# Accelerating Diverse Cell-Based Therapies Through Scalable Design

**DOI:** 10.1146/annurev-chembioeng-100722-121610

**Published:** 2024-07-03

**Authors:** Emma L. Peterman, Deon S. Ploessl, Kate E. Galloway

**Affiliations:** Department of Chemical Engineering, Massachusetts Institute of Technology, Cambridge, Massachusetts, USA

**Keywords:** cellular engineering, stem cells, biomanufacturing, genome editing, cell-based therapies, synthetic biology

## Abstract

Augmenting cells with novel, genetically encoded functions will support therapies that expand beyond natural capacity for immune surveillance and tissue regeneration. However, engineering cells at scale with transgenic cargoes remains a challenge in realizing the potential of cell-based therapies. In this review, we introduce a range of applications for engineering primary cells and stem cells for cell-based therapies. We highlight tools and advances that have launched mammalian cell engineering from bioproduction to precision editing of therapeutically relevant cells. Additionally, we examine how transgenesis methods and genetic cargo designs can be tailored for performance. Altogether, we offer a vision for accelerating the translation of innovative cell-based therapies by harnessing diverse cell types, integrating the expanding array of synthetic biology tools, and building cellular tools through advanced genome writing techniques.

## 1. ENGINEERING CELLS WITH GENETICALLY ENCODED FUNCTIONS

Cells form the central information processing units of tissues, enabling coordinated functions across a variety of length and timescales. Integrating signals from their biochemical and physical environments, cells perform complex processing that supports spatiotemporal behaviors (1). The durability of continuous monitoring via cells makes them attractive chassis for long-term surveillance of disease states and repair of tissue. Although some human tissues regenerate, the heart and central nervous system display particularly limited regenerative capacity (2, 3). Consequently, replacement of cells offers a promising treatment for patients suffering from trauma and disease, representing one of the most ambitious goals of cell engineering.

**Figure 1:**
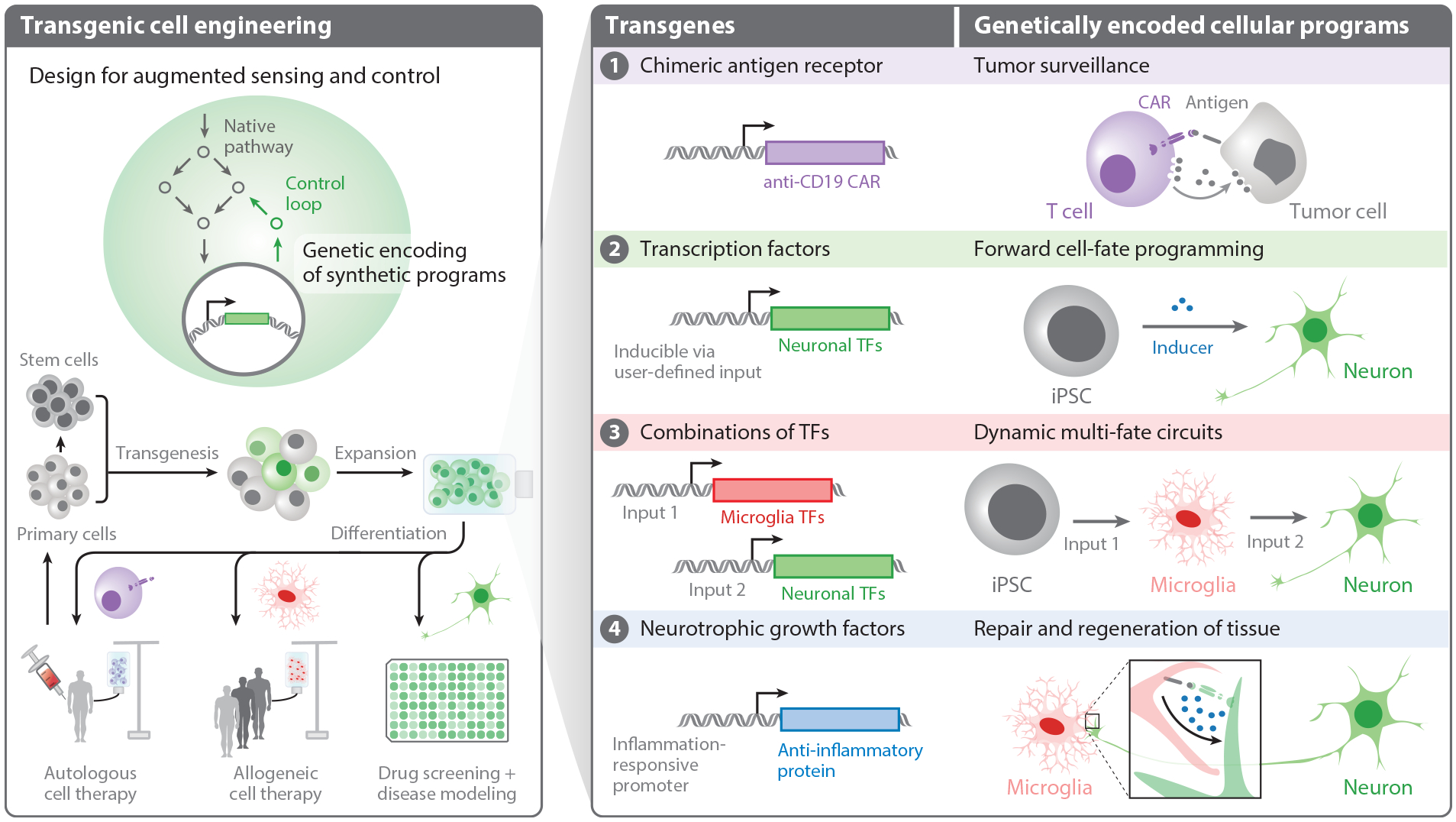
Transgenes integrate into native biological processes, pathways, and networks to augment the capabilities of cells and generate novel genetically encoded programs. Native pathways enable cells to transmit extracellular signaling into cellular responses that include differentiation, proliferation, and death. Addition of transgenes and transgenic controllers can augment or reprogram these endogenous networks for applications in drug screening, disease modeling, and cell therapies. Transgenes can encode a wide variety of genetic programs. ① Synthetic receptors such as CARs program T cells to identify and kill tumor cells (36, 37, 69, 102, 103, 114, 149). ② Overexpression of TFs induces cells to transition from one identity to another (186). Forward programming employs the overexpression of cell type–specific TFs to direct iPSCs in transitioning to the associated cell type (187). ③ Dynamic, multi-input circuits allow temporally staged induction of cell fates, enabling specific cellular functions and sequence of functions from a genetically homogeneous population of cells (188). By engineering cells that naturally surveil and respond to damage, theranostic circuits can be used to target repair and regeneration of tissue at the site of damage.④ In particular, microglia are well-suited for surveillance, intervention, and repair in the central nervous system (189–193). Abbreviations: CAR, chimeric antigen receptor; iPSC, induced pluripotent stem cell; TF, transcription factor.

To realize the potential of cell engineering, both genetic and nongenetic methods of controlling cells have been explored. Nongenetic methods rely primarily on the instructive cues of biomolecules in the extracellular space to program cells. These signals act through native sense-and-respond pathways to adjust cellular behavior and fate for applications like differentiation of pluripotent stem cells (4–8). Additionally, physical and electromagnetic forces can influence transcriptional networks via signaling pathways (9). While control of these physiochemical inputs can direct cellular responses, extracellular programming relies on native pathways and requires strict control of the extracellular environment to induce changes in cellular behavior through transient stimuli. As a result, extracellular programming offers limited performance for in vivo applications that require durable sense-and-respond functions. Alternatively, genetic programming via the introduction of transgenes expands the range of behaviors accessible to cells (10–13). Transgenesis introduces genes to host cells to directly repair or augment the native functions of cells through interaction of transgenes with native cellular pathways (Figure 1). Transgenes can encode native genes outside their normal genomic contexts, subjecting the transgene to different transcriptional and post-transcriptional regulation compared to its native counterpart. This attribute permits exploration of novel cellular states not accessible in endogenous contexts.

In 2000, watershed synthetic biology papers on the repressilator (14) and toggle switch (15) demonstrated that synthetic transgenic systems could program specific dynamic responses, offering a vision for the future of transgenic control in living cells. Engineering mammalian cells with transgenes offers a wide range of potential therapeutic applications (16, 17) (Figure 1). Noteworthy examples of programmed functions in mammalian cells include tumor surveillance, enzyme production, enzyme homeostasis, and anti-inflammatory response (18–24). Toward the goal of precisely programming mammalian cell behavior, synthetic biologists have designed and optimized many sophisticated genetic circuits, including multistable switches (25, 26), feedforward loops (27, 28), and integral controllers (29). In theory, the design of modular genetic cargoes will support plug-and-play frameworks and the encoding of more sophisticated, dynamic cellular behaviors. However, the difficulty in predictably engineering cells has limited the translation of genetic controllers beyond the lab scale (30, 31). Because predictable design remains challenging, engineering robust behaviors in mammalian cells often requires labor-intensive, iterative derivation of monoclonal cell lines. The increased timeline, costs, and mutation risk associated with prolonged in vitro culture coupled with the sensitivity of primary cells to harsh single-cell isolation conditions render such methods infeasible for most primary cells (32).

Various application-specific constraints exist in cell engineering, including engineering timelines, in vitro cell lifetimes, and safety regulations. Thus, no single engineering approach works for every application. Here, we highlight the recent advances in mammalian cell engineering that guide the selection of engineering strategies for a specific application. This review is structured in three parts. First, we survey mammalian cell engineering applications and the associated challenges of engineering specific cell types for these applications. Second, we examine genomic integration tools. Third, we review design criteria and methods for optimizing genetic cargoes for a given integration technique. Finally, we conclude with a vision for the future of mammalian cell engineering.

## 2. CELL ENGINEERING APPLICATIONS AND ENGINEERING SPECIFICATIONS

**Figure 2:**
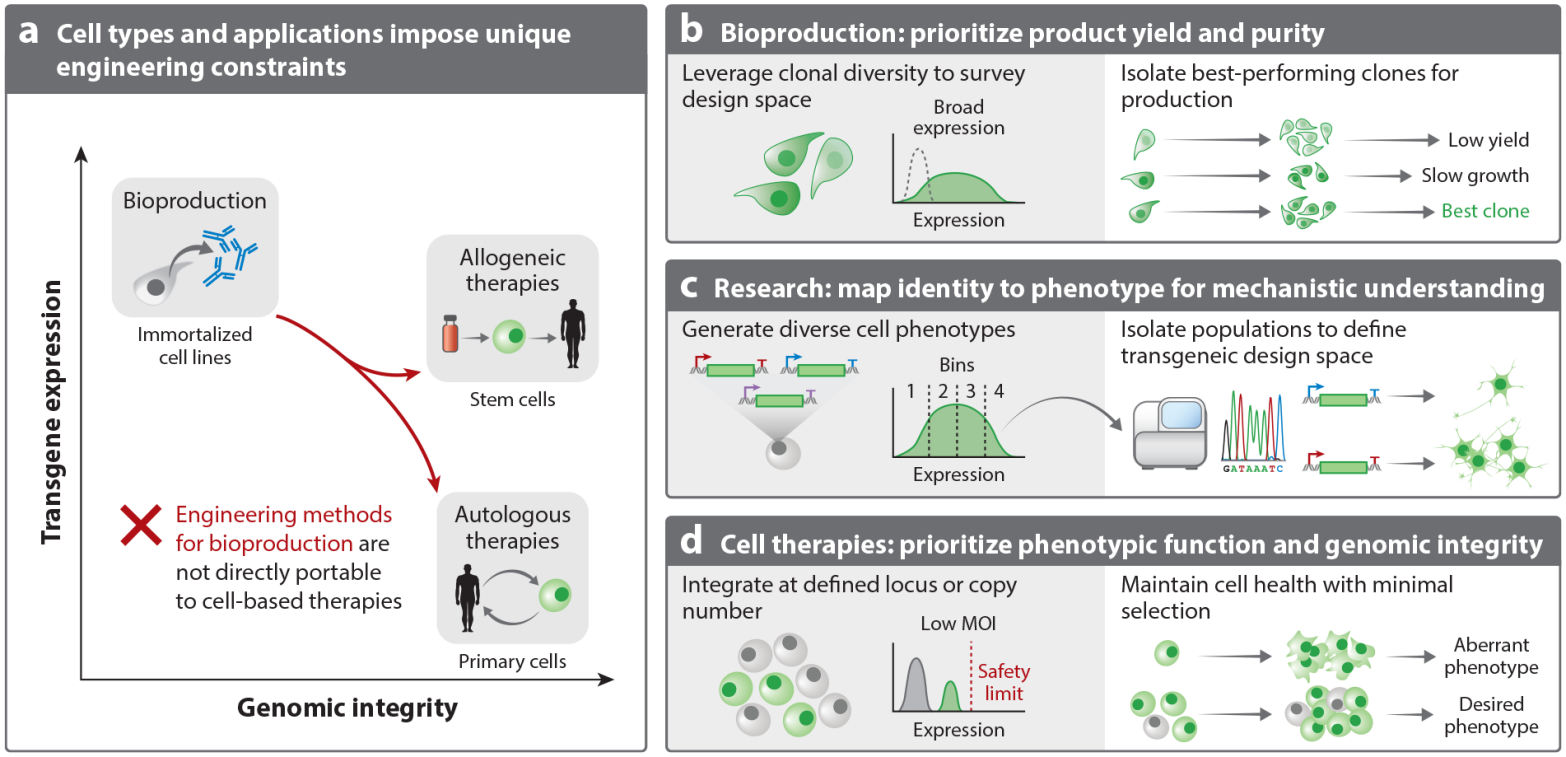
Overview of cell engineering applications and constraints for optimizing performance. (*a*) The cell types relevant for different cell engineering applications place unique constraints on which methods and characterization can be performed. The earliest engineering methods were developed for bioproduction and prioritize product yield and purity over genomic identity. (*b*) As a result, bioproduction methods rapidly generate populations with large phenotypic diversity and rely on monoclonal selection to produce cell lines with high production capacity. These methods are not directly portable to the production of cell therapies, which require more stringent maintenance of genomic identity. (*c*) Research applications often leverage techniques to create clonal diversity and use this diversity to map genotype to phenotype. (*d*) Autologous therapies with primary cells must limit the copy number of integrated transgenes to reduce toxicity associated with high transgene expression. Low multiplicity of infection (MOI) limits integration copy number and transgene expression. These therapies introduce minimal selection workflows to maintain cell health and support in vivo efficacy. New techniques for cell engineering and for cargo design are needed to translate progress in synthetic biology to clinical contexts.

Historically, mammalian cell engineering has focused on harnessing immortalized cell lines, such as Chinese hamster ovary cells and human embryonic kidney cells, as producers of pharmaceutical products synthesized from biological sources, referred to as biologics (33). For efficacy and safety, many protein products require specific post-translational modifications, which microbial cells cannot perform (34). Consequently, 85% of the novel biopharmaceuticals approved since 2018 are manufactured using mammalian cells (35). Accordingly, the engineering pipeline for producer lines optimizes for high transgene expression to maximize yield of the target biologic (Figure 2a). Long timelines for offline optimization remain feasible for biologic production because they employ immortalized cell lines amenable to harsh selection and high-efficiency transgene integration methods. However, the limited lifetime of most primary cells renders workflows with expanded culturing times infeasible.

Engineered mammalian cells have expanded as products for cell-based therapies, drug discovery, and basic research (16). Over the last decade, clinical trials for chimeric antigen receptor (CAR) T cell therapies (36, 37) and stem cell–based therapies (16) have demonstrated safety and efficacy in treating diverse diseases. However, these applications often require engineering of patient-derived primary cells, whose shorter in vitro lifetimes and greater sensitivity to culture conditions make scale-up challenging (Figure 2a). Given the constraints imposed by the cell type and safety criteria, the engineering techniques optimized for biologic production do not translate to most cellular therapeutics. Here, we detail the challenges associated with engineering mammalian cells for therapies, highlighting the role research plays in bridging the gap between biologic production and the next generation of engineered cell–based products.

### 2.1. History of Mammalian Cell Engineering for Biologic Production

**Figure 3:**
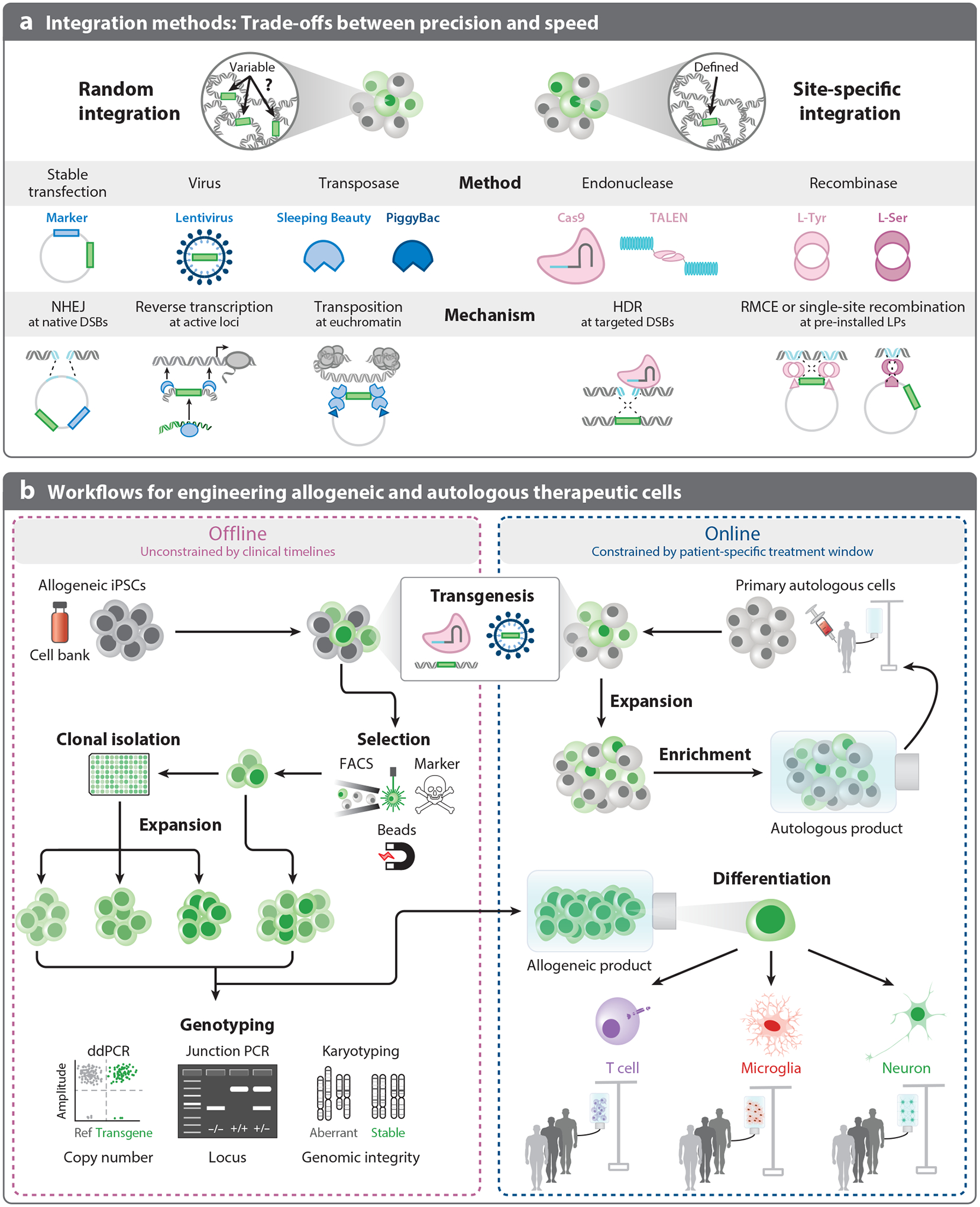
Overview of transgenesis methods and workflows to engineer cells for specific applications. (*a*) Selection of random or site-specific integration methods depends on the cell type and application. Stable transfection allows integration via a coupled transgene–marker gene pair that integrates randomly through native DSBs via NHEJ. Moderately sized cargoes can be integrated via viral vectors such as retroviruses and lentiviruses. Transposons such as Sleeping Beauty and PiggyBac enable the integration of large payloads in a broad range of clinically relevant cell types. The lack of control over the transgene’s insertion site and copy number generates clonal diversity. Site-specific integration via programmable endonucleases through HDR presents a more precise and potentially safer route of transgenesis. CRISPR/TALENs have been leveraged to engineer LPs harboring tyrosine/serine recombinase recognition sites at a user-defined locus. LPs enable swift integration of transgenes via RMCE or single-site insertion. However, the precision of targeted methods requires HDR and extended culture times to isolate and expand the engineered line, which is impractical for primary and postmitotic cells. (*b*) Due to patient needs and the use of primary cells, many autologous cell therapies have severe constraints on production timelines, with limited offline optimization. Considering the most sensitive applications would benefit most from precision techniques, iPSCs could bridge this critical divide between portability and precision. iPSCs are amenable to extended cell line engineering workflows, allowing for more offline optimization and selection. Engineering allogeneic iPSCs with clinical-grade product standards would realize the potential of precision genetic engineering and usher in the next generation of cell therapies. Abbreviations: ddPCR, droplet digital PCR; DSB, double strand break; FACS, fluorescence-activated cell sorting; HDR, homology-directed repair; iPSC, induced pluripotent stem cell; LP, landing pad; NHEJ, nonhomologous end joining; PCR, polymerase chain reaction; RMCE, recombinase-mediated cassette exchange.

The field of biologic production relies on cell lines that are fast growing, amenable to suspension culture, and adaptable to dynamic conditions throughout the manufacturing life cycle. Immortalized cell lines such as Chinese hamster ovary and human embryonic kidney cells enable the exploration of diverse phenotypic landscapes (Figure 2b,c). Although the genomic plasticity of these cells facilitates rapid cell engineering, it also potentiates massive epigenetic changes (38) and genomic rearrangements (39) because the combined effects of metabolic burden and natural selection exacerbate genomic instability (40). Traditionally, genomic instability has been overcome by brute-force offline engineering methods. Combining rapid random integration and enrichment with antibiotic-based selection, productive lines can be identified through screening of large monoclonal libraries (Figures 2b and 3a). Although clone identification can take up to six to nine months to establish a production line (41), the costs of this extensive optimization can be recuperated over time because immortalized producer lines provide a durable supply of products for years.

### 2.2. Engineering Cells for Fundamental Research

Disease modeling, therapeutic development, and gene circuit prototyping often rely on cell engineering. For these applications, cell engineering supports mapping of genomic and genetic variation to changes in cell states and fates (Figure 2c). Random integration techniques generate diverse starting populations, allowing exploration of the genetic design space (42). The development of new high-throughput characterization methods that use single-cell RNA sequencing has accelerated the mapping of cell phenotypes to genotypes and to nongenetic determinants of fate (43, 44) (Figure 2c).

Even with precise, site-specific transgene integration, significant differences in expression levels can manifest between clones due to clonal variation at the genetic and/or epigenetic levels (45), as well as due to stochastic fluctuations (43, 46, 47). To eliminate genetic variation, gene circuit designs and synthetic biology tools are often characterized in monoclonal cell lines (48, 49). For short-timescale phenomena such as transcriptional bursting, monoclonal selection eliminates confounding sources of clonal diversity that obscure mechanistic understanding (50). Therefore, monoclonality remains useful across integration methods (Figure 3a).

Even within genetically homogeneous populations, cells can exhibit distinct, heritable differences. Cellular reprogramming and oncogenesis provide an intriguing test bed system for understanding nongenetic sources of variation that contribute to diverging cell fates (43, 46, 51–53). For disease modeling, clone tracing strategies can identify rare clones and pathways exploited in the development of resistance (54). By introducing transgenic reporters and circuits into stem cells, these transgenic systems report on cell-specific patterns of expression in healthy and diseased contexts. For more discussion on the use of transgenic systems for disease modeling and drug discovery, we refer the reader to Beitz et al.’s (55) extensive review of synthetic gene circuits as tools for drug discovery. Overall, research applications of cell engineering employ highly variable engineering methods to quickly survey the available design space. However, researchers must also rely on single-cell isolation or lineage tracing to identify which genetic factors contribute to the resulting population variability and leverage this information to develop better methods for translational applications like cell therapies.

By understanding genetic and nongenetic sources of variability, researchers can design and build genetic control systems that perform amid these emergent disturbances. By solving these challenges in cell lines, fundamental research will provide the insights needed to bridge the technical gap between methods designed for biologic production and those that can perform in cell-based therapies.

### 2.3. Engineering Cells for Therapies

As the number of trials and approved cell-based therapies expands rapidly (56, 57), our ability to treat the growing number of patients will become limited by inefficient manufacturing pipelines. Although the success of CAR-T clinical trials and the recent US Food and Drug Administration (FDA) approval of a cell-based treatment for type I diabetes (58) highlight the therapeutic potential of engineered cells, engineering of autologous (i.e., patient-specific) therapies requires extensive resources, challenging logistics, and extended time frames to generate a product that serves only a single patient (59, 60). The associated costs of autologous therapies (~$500,000) limit patient access to otherwise lifesaving medicines (61). Due to risks of tumor formation and immune rejection, cell-based therapies are subject to stringent quality-control metrics (62), and manufacturing processes must prioritize genomic integrity of the final cell product (Figure 2d). Consequently, the techniques developed and optimized for biologic production do not translate to cell-based products.

Autologous therapies rely on patient-derived primary cells. Because many applications require short production timelines, the extent of genetic engineering must be limited and robust (Figure 3b). Autologous therapies demand engineering methods and genetic cargo designs that function reliably in polyclonal cell populations, minimizing the length of selection required to produce a safe, effective product. Even simple transduction of transgenes remains limiting for engineering primary cells. Furthermore, comorbidities originating from sick hosts can render autologous cell therapies resistant to manipulation and ex vivo expansion (63), leading to insufficient (64) and/or aberrant (65) therapeutic activity in vivo.

Alternatively, off-the-shelf allogeneic therapies provide a product suitable for a multitude of patients in an expedited manner by eliminating the need to engineer a patient-specific starting population of primary cells (17, 66). Induced pluripotent stem cells (iPSCs) represent a privileged allogeneic cell source because iPSCs are expandable, amenable to gene editing, and differentiable into many cell types (67). Thus, a single master iPSC line offers the capacity to generate large quantities of rare cell types engineered with prosthetic and theranostic functions. In addition to lowering cost (68), an allogeneic pipeline grants manufacturers the time to screen offline for pristine cells from healthy donors and fine-tune transgenic designs for a defined genetic background (Figure 3b). The more rigorous offline engineering capabilities allowed by an allogeneic approach may increase therapeutic activity, immunocompatibility, and safety profiles.

While academia and industry transition toward a more scalable allogeneic cell therapy market (69, 70), expansion of existing autologous cell therapies would benefit from high-efficiency transgene integration techniques, ensuring a large fraction of isolated primary cells are transduced. Additionally, methods that minimize cellular stress, reduce variability in polyclonal populations, and support online screening can increase the efficacy of both autologous and allogeneic therapies.

## 3. MAMMALIAN CELL ENGINEERING TOOL KIT

Regardless of cell source, the process of transgenesis remains relatively inefficient and highly variable. To support scalable pipelines for generating transgenic stem cells, we need efficient integration of transgenic cargoes and sustained expression of transgenes in the desired cell type. Critically for iPSC-derived cells, transgenes must not silence during or after differentiation (31).

Random and site-specific methods for integrating transgenes offer different advantages in terms of speed, precision, and flexibility. Cell engineering pipelines must equip cells with fine-tuned functions while balancing the need for rapid, scalable production. Delivery of more sophisticated genome editing tools and genetic cargoes often reduces the speed and efficiency of transgenesis. Below, we highlight the capabilities and limitations of various transgenesis approaches for integration of full transcriptional units. For methods of modifying endogenous loci through gene knockout, tagging, or mutagenesis, we refer readers to a review of genome editing tools (71). Additionally, genetic cargo delivery methods can have significant impacts on engineering efficiency, and we refer readers to dedicated reviews on this topic (72–74).

### 3.1. Random Integration Techniques

Prior to the development of programmable nucleases, scientists relied on random integration methods to insert transgenes portably and reliably. Stable transfection is the simplest of these random techniques, requiring only a selection marker, and has long served as the workhorse for integrating transgenic DNA cargoes for bioproduction. The subsequent development of viral vectors and transposon systems has expanded the range of cell types in which transgenes can be integrated efficiently. Notably, a wide array of clinical trials and FDA-approved cell products leverage high-efficiency viral delivery to integrate therapeutically relevant cargoes into primary cells (1, 75, 76).

The simplicity of random integration comes at the expense of inherent variability. Random integration lacks control over the vector copy number (VCN) and the site(s) of transgene integration, which may lead to unpredictable cell behaviors (Figure 3a). The inability to control VCN and prevent insertional mutagenesis may obscure the characterization of individual genetic parts, such as promoter strength, and inject bias in genome-wide screens (77). As a result, random integration methods often accompany monoclonal selection in bioproduction and research applications. In the context of cell therapies, variable transgene expression associated with integration loci may increase or decrease expression of the transgene and impact therapeutic efficacy. For example, excessive CAR expression can drive T cell exhaustion, excessive cytokine release, and toxicity (78–80). Moreover, insertions that disrupt tumor suppressors or upregulate oncogenes may transform cell products. Although no formal upper limit of VCN for gene and cell therapies exists, the FDA recommends a VCN <5 to minimize oncogenesis (81) (Figure 2d). Developing methods that yield predictable VCNs early in cell manufacturing may improve scalability and the performance of engineered cells (82).

#### 3.1.1. Stable transfection.

Stable transfection exploits spontaneous DNA double-stand breaks (DSBs) to insert transgenes (Figure 3a). Mammalian cells most efficiently repair DSBs via nonhomologous end joining (NHEJ), providing an access point for the insertion of foreign DNA (83). Stable clones are generated by transfecting DNA encoding the desired transgene alongside a selection marker, often encoding resistance to an antibiotic such as neomycin (25). Extended selection for a period of weeks yields a polyclonal population that has integrated the transfected DNA at random DSB sites. The methionine sulfoximine selection system serves as a classical gene amplification system to enrich for clones with increased transgenic expression (84, 85). The resulting clonal diversity supports downstream screening and identification of stable, high-expressing clones.

Although time-consuming and laborious, the simplicity of stable transfection and gene amplification yields productive platform lines and serves as a primary method for the production of biologics (86, 87). Stable transfection and massive library screening work well in bioproduction applications, but the harsh and lengthy culturing conditions required to identify stable, high-expressing clones adapt poorly to primary cells with finite passage numbers. Moreover, the infrastructure to screen hundreds to thousands of monoclones remains limited.

#### 3.1.2. Retroviral vector–based methods.

Viruses readily integrate foreign DNA in the cells they infect, motivating efforts to repurpose viruses as transgenic engineering tools. Engineering efforts focused initially on the *Retroviridae* family of viruses, including *γ*-retroviral-derived and HIV-1-derived vectors (88). Retroviruses encode their genomes on single-stranded RNA. Upon infection, viral reverse transcriptase and integrase catalyze reverse transcription and integration of proviral DNA into the host genome (88) (Figure 3a). Through removal of viral genes required for replication, replication-incompetent viral vectors with enhanced safety profiles were developed (89, 90). In 1990, the first clinical trial involving recombinant retroviral gene therapy delivered adenosine deaminase for the effective treatment of severe combined immune deficiency (91). However, side effects arising in a subset of patients from this clinical trial drove efforts to improve vector safety. The following decades witnessed immense strides in modifying retroviruses for cell engineering.

Because retroviruses can effectively integrate transgenic cargoes up to approximately 8 kb, the FDA-approved CAR-T therapies Yescarta and Tecartus leverage *γ*-retroviral vectors to deliver the CAR transgene (75). Despite clinical success, the transcriptional activity of *γ*-retroviral long terminal repeats can potently activate proto-oncogenes (92). The propensity for insertion at transcriptional start sites may exacerbate the potential for insertional mutagenesis (93). Although enhanced screening of engineered cells may mitigate these dangers, reports of oncogenesis in *γ*-retroviral human gene therapy trials have led to strict safety regulations for retrovirally engineered cell products (94–96). Currently, *γ*-retroviral vectors find extensive use as research tools.

The heightened concerns over safety and tropism limitations of *γ*-retroviral vectors spurred the development of HIV-1-derived lentiviruses (97), which can transduce postmitotic and quiescent cells (76). Broader tropism enables the exploration of gene function studies in postmitotic cell types, including neurons (98) and cardiomyocytes (99). Like *γ*-retroviral vectors, lentiviral vectors have gone through multiple design cycles, with newer generations including self-inactivating design, partitioning viral genes across multiple plasmids, and disruption of transcriptional activities of long terminal repeats (100). These efforts culminated in clinical success, with the FDA-approved CAR-T therapies Kymriah, Breyanzi, and Abecma (101) all leveraging lentiviral delivery of the CAR payload (Figure 3b). However, like *γ*-retroviral vectors, lentiviruses show insertional bias within transcriptional units (100), imposing limits on VCN to mitigate the risk of insertional mutagenesis. Additionally, efficient production of multigene lentiviral vectors remains nontrivial, making it difficult to integrate complex genetic modules reliably with viral methods.

#### 3.1.3. Transposase-based methods.

Like viral integrases, transposases catalyze excision of DNA sequences flanked with transposase-specific inverted terminal repeats and insertion into chromosomal DNA. The most notable synthetic transposon systems, Sleeping Beauty and PiggyBac, have demonstrated promise in clinical settings (102, 103). Transposons accommodate substantially larger payloads (more than 100 kb) compared to retro- or lentiviruses. Compared to viral vectors, transposase methods are simpler to prepare. Transgenesis requires only the transposase, encoded as either plasmid DNA or messenger RNA (mRNA), and the transgene flanked by the cognate inverted terminal repeat sequences. However, nonviral delivery of DNA remains a challenging roadblock to engineering many cell types via transposases (104).

Despite their advantages, like all random integration methods, transposon vectors potentiate insertional mutagenesis. Whereas Sleeping Beauty exhibits a close-to-random integration profile with a slight bias for integration into gene bodies (105), PiggyBac demonstrates a clear bias for transcriptional start sites and CpG islands (106). Although these insertional biases suggest a more favorable safety profile for Sleeping Beauty (107), transposons carry the risk of subsequent transposition following the initial integration (108). Multiple transposition events may occur as the transgene hops to different loci, which may induce large-scale chromosomal rearrangement and compromise genomic integrity. Minimizing transposase expression, either with self-inactivating DNA vectors (109) or by supplying the transposase as mRNA (110), can mitigate these risks.

### 3.2. Site-Specific Integration Techniques

Site-specific integration offers precise installation of transgenes and the potential to eliminate noise introduced by variation in locus and VCN. The development of zinc-finger nucleases (ZFNs) and transcription activator–like effector nucleases (TALENs) resulted in a paradigm shift toward precision genome modification with programmable nucleases (111). The establishment of guide-programmable nucleases, such as the CRISPR/Cas system, rapidly expanded the speed and scale of targeted genome editing and transgenesis (112). With the ability to target virtually any genomic location, transgenes could be placed in regions of the genome that support stable transgene expression without adversely impacting endogenous expression. Accordingly, pipelines to target such genomic safe harbors (GSHs) exist in immortalized cell lines (113), primary cells (114), and iPSCs (115). Although GSHs are particularly useful for specifying sites for efficient transgene integration, the stability of transgene expression at GSHs remains variable across cells and time in culture.

In cell types amenable to extended cultivation and single-cell cloning, engineering landing-pad (LP) lines provides a scalable means to introduce transgenes and prototype integrated genetic circuits while controlling for locus effects and copy number (116). LPs leverage CRISPR/TALEN-mediated homology-directed repair (HDR) to install recombinase acceptor sites, permitting the subsequent use of more efficient recombinase-based methods to site-specifically integrate transgenes in parallel. LPs have served as potent tools in producer cell line development (45, 117), iPSC engineering (118), and disease modeling (119). The precision gained with site-specific methods comes at the cost of efficiency, as mammalian cells predominantly employ NHEJ to fix DSBs (120). Nevertheless, site-specific integration offers enhanced polyclonal performance compared to random integration, potentially bypassing the intractable derivation of monoclonal cell lines in generating engineered cell products (Figure 3b).

#### 3.2.1. Endonuclease-based methods.

ZFNs conceptualized the notion of fusing DNA-binding domains and nucleases, combining an array of DNA-binding zinc fingers with a FokI nuclease to specify the site of DNA cleavage. After cleavage, transgenes can integrate at the site of the programmed DSB via HDR using sequences homologous to the region flanking the DSB. Whereas ZFNs offered a vision for site-specific integration, TALENs streamlined the programmable design of the DNA-binding domain through modular tiling of peptide arrays that recognize the desired target DNA sequence (Figure 3a). The enhanced programmability led to TALENs being named “Method of the Year” by *Nature* in 2011 (121). However, the CRISPR/Cas9 editing system quickly supplanted TALEN-based editing (122).

Unlike TALENs, which require the design and validation of a novel protein for each genomic target, the Cas9 nuclease is directed to genomic targets by a programmable 20-bp RNA sequence encoded on a single guide RNA (Figure 3a). Whereas TALEN-based editing has a higher specificity with fewer off-target DSBs, CRISPR offers simpler programmability of the target site (123). Moreover, whereas TALENs display superior editing at heterochromatin (124), euchromatin remains a more common target for transgene integration. As a result, the last decade has seen concerted efforts to enhance CRISPR editing efficiency. The precision and portability of CRISPR have increased through repurposing of other CRISPR systems, such as Cas12a, as well as protein engineering of Cas nucleases with enhanced targeting fidelities (125), novel protospacer adjacent motif sites (126), and reduced sizes (127, 128). For applications in cell therapies, the integration of CAR payloads into the endogenous *TRAC* locus in T cells underscores the therapeutic potential of CRISPR-mediated transgene insertion because this strategy simultaneously optimizes expression and improves immunological compatibility (69).

Following DSB induction, cells can repair DNA through NHEJ or HDR. A given cell type’s preference for either repair pathway significantly impacts successful editing frequencies. Because NHEJ competes with HDR, NHEJ impedes HDR-mediated integration of transgenes. Moreover, HDR functions during the S and G2 phases of the cell cycle, limiting integration in postmitotic cell types. The criticality of HDR in successful targeted integration has led to many methods to boost HDR, including addition of small molecules that either inhibit NHEJ or increase HDR (129), interventions to synchronize cell cycle in S/G2 (130), biomolecular designs that tether Cas9 to HDR machinery or to the transgene donor DNA (131, 132), and addition of chemical modification of the transgene donor DNA (133). Even with these improvements, integrating transgenes via HDR still requires lengthy selection and often cumbersome genotyping screens to enrich and isolate correctly edited clones. Moreover, CRISPR introduces the risk of off-target effects, ranging from small mutations (134) to large-scale chromosomal aberrations (135). Finally, CRISPR genotoxicity elicits a DNA damage response capable of cell cycle arrest (136), biasing the expansion of clones with oncogenic mutations (137).

#### 3.2.2. Recombinase-based methods.

Compared to HDR machinery, site-specific recombinases (SSRs) efficiently exchange homologous DNA sequences. Classically used for constructing conditional knockout cassettes in transgenic mice (138), the tyrosine recombinases Cre and Flp can be used for transgene integration via a SSR recognition site, as used in LP lines. A SSR recognition site is first integrated in the genome via endonuclease-based HDR integration. Once established in a LP line, the SSR recognition site can be used to integrate transgenic cargoes via the corresponding SSR (Figure 3a). LPs have facilitated rapid, site-specific integration of transgenes in various immortalized cell lines (45, 116, 117, 139) and stem cells (119, 140, 141).

In addition to tyrosine recombinases, large serine recombinases (LSRs) such as Bxb1 and PhiC31 have emerged as potent genome editing tools (142–144). Compared to the reversible recombination reactions of tyrosine recombinases, LSRs catalyze unidirectional reactions, which support permanent genomic alterations. Moreover, LSRs accommodate larger transgenic cargoes, with an undefined upper limit (143). Bxb1 has proven especially useful because its fusion to a Cas9 prime editor consolidates the typical two-step LP line creation and recombinase-mediated transgene integration pipeline. Once delivered, the fusion genome editor can both install a Bxb1 recognition site and mediate recombination to integrate the transgene (144).

The exact mechanism of transgene integration via SSRs depends on the LP architecture. Whereas a single SSR site integrates the entire donor vector, two orthogonal SSR sites integrate payloads flanked by the cognate acceptor sites via recombinase-mediated cassette exchange (Figure 3a). While the single-site method permits more efficient integration of larger payloads, recombinase-mediated cassette exchange bypasses the co-integration of unwanted plasmid sequences on the vector, which may influence transgene expression and elicit immunogenicity from bacterial DNA sequences (145). If using a cell type amenable to creation of a LP line, recombinase-based methods allow for rapid cell engineering with lower oncogenesis risk.

#### 3.2.3. Genomic safe harbors.

With programmable endonucleases’ ability to target essentially any region in a genome, the question becomes where one should integrate transgenes. Ideally, GSHs offer sites amenable to integration, support stable transgene expression, and insulate crosstalk with the rest of the genome. To date, there exist a handful of consensus GSHs in the human genome, including *AAVS1, CLYBL*, and *CCR5*, often located in introns of nonessential genes (146–148). Although the predominantly used *AAVS1* GSH can support expression of transgenes in several cell types (115, 149), silencing at the *AAVS1 locus* is well-documented (150, 151). In addition to transgene silencing, expression from *AAVS1* can vary based on cargo design. Integration at *AAVS1* places transgenes within an intron of *PPP1R12C*, which could result in transcriptional interference from the endogenous promoter. Supportive of this hypothesis is the recent observation that orienting transgenes in either convergent or tandem syntaxes at *AAVS1* yielded different expression profiles (150). Critical for engineering stem cells, silencing at *AAVS1* occurs after differentiation of iPSCs for multiple cell lineages, including myeloid cells (152), hepatocytes (153), and cardiomyocytes (151). Finally, *AAVS1* resides in a gene-rich location, with integrated payloads reported to disturb adjacent gene expression (146). Collectively, the unpredictable expression of transgenes at *AAVS1* limits its full utility as a GSH for engineering stem cells.

Limits in existing GSHs have motivated the search for alternative sites (146). Recently, a bioinformatics-guided approach identified region optimal for gene insertions 1 and 2 (Rogi1 and Rogi2) (147). Located more than 50 kb away from known coding DNA sequences and regulatory elements, Rogi1 and Rogi2 mediated higher and more stable transgene expression than *AAVS1* in immortalized cell lines and primary cells. Moreover, transgene expression from Rogi1 or Rogi2 did not impact the global transcriptome, indicating that Rogi1 and Rogi2 support seamless integration of synthetic circuits within host cells. Whereas initial efforts focused on identifying GSHs by primary DNA sequence, recent efforts to identify GSHs have integrated epigenetic and 3D-chromatin data to elucidate cell-type-specific loci that reside in euchromatin and do not interact with tumor suppressor/oncogenes (146). Higher-resolution genomics techniques will continue to inform logical GSH selection.

Current integration methods often offer trade-offs between efficiency and precision. For example, high-precision methods like CRISPR/TALEN-mediated integration may yield better safety profiles for autologous cell therapies, but higher-efficiency transgenesis has favored viral integration for autologous therapies (Figure 3b). The expansion of methods to robustly produce hypoimmune allogeneic cells from iPSCs (67, 154, 155) will increase the number of cell products brought to the clinic. Incorporating offline engineering techniques for allogeneic cells will support the quality and quantity of cells required to meet the demand for cell-based therapeutics. Although integration methods provide a range of options for engineering cells, each application requires the co-design of integration method and genetic cargo to maximize efficiency and safety.

## 4. DESIGN AND OPTIMIZATION OF GENETIC CARGOES

Achieving stable performance of genetic programs requires coordinated design of integration methods and cargoes. Ideally for clinical translation, genetic modules can be swiftly integrated into the genome to yield polyclonal cell lines that perform robustly in noisy physiological environments. Reliance on time-intensive methods has limited the adoption of synthetic genetic programs into cell-based therapies (41). Well-designed cargoes should harness the most efficient transgenesis methods and mitigate sources of noise that impede function.

**Figure 4:**
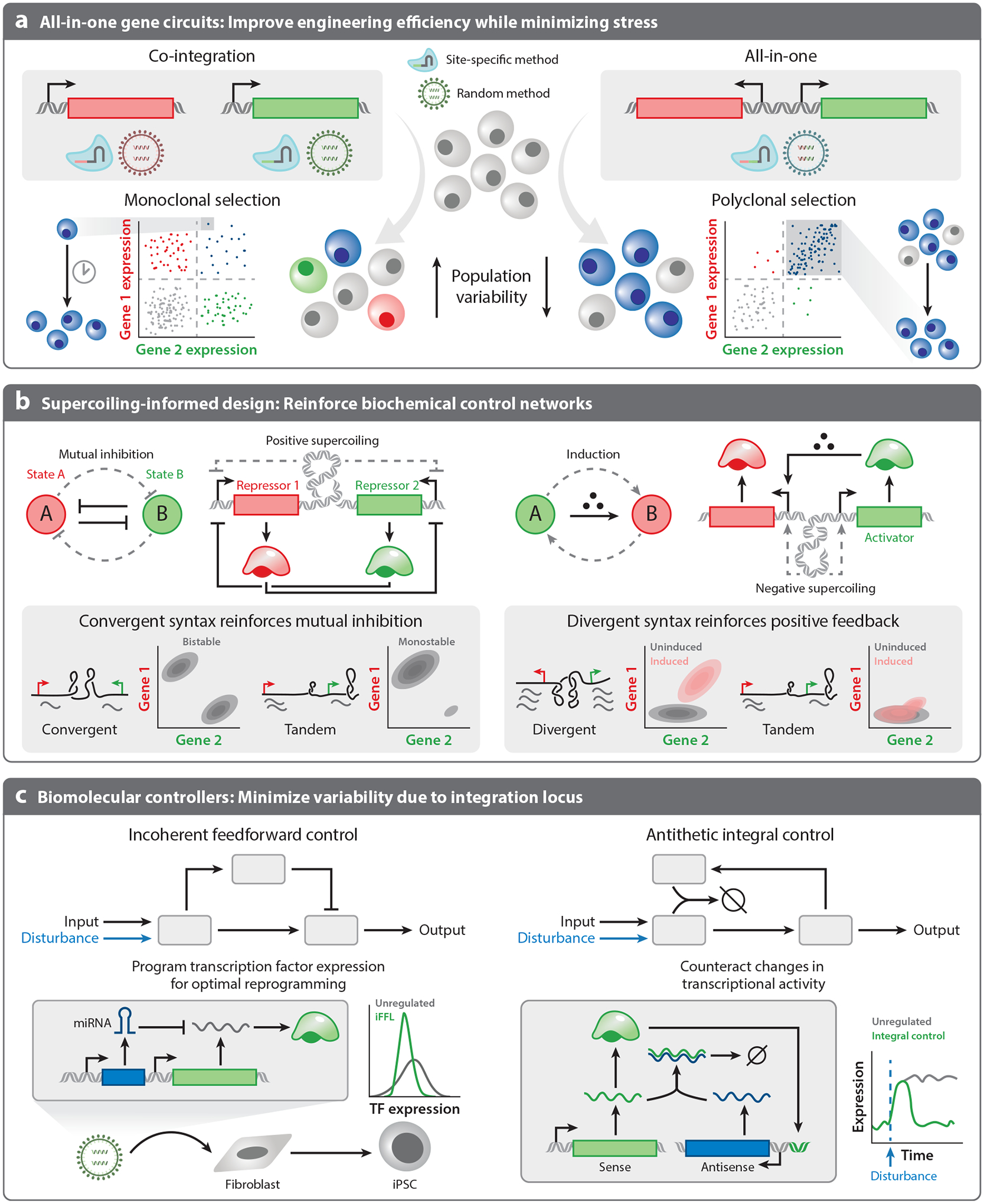
Tuning genetic cargo design to improve performance of transgenic systems. The design of genetic cargoes contributes to the expression profile of each transgene. (*a*) Engineering all-in-one genetic cargoes can increase performance of both site-specific and random integration methods. All-in-one vectors require only a single integration step, resulting in increased engineering efficiency while reducing genotoxic stress. Additionally, all-in-one vectors may enhance polyclonal performance, reducing the need for lengthy monoclonal selection and outgrowth steps. (*b*) Transcription of genes in close proximity to each other can be coupled due to supercoiling-mediated feedback, and this feedback (*dashed lines*) can be harnessed to reinforce desired biochemical control networks in compact gene circuits (161). Convergent gene syntax is predicted to reinforce mutual inhibition, crucial for proper functioning of multistable circuits like the repressor-based circuit that maintains two stable states, State A and State B, marked by gene 1 and gene 2, respectively. Bistability is achieved through mutual inhibition meditated by repressors and DNA supercoiling. Divergent syntax is predicted to reinforce positive feedback (*dashed lines*). Expression of the activator and regulated gene should increase as the small molecule is added due to the positive feedback from negative supercoiling that should increase the rate of polymerase binding to both promoters. (*c*) Variation in expression can be reduced using compact biomolecular controllers. Controllers can reduce the variability of proteins that may result from variations in copy number and integration locus. For example, a miRNA-based iFFL controller can limit variation and control transcription factor expression during fibroblast to iPSC reprogramming (172). An integral controller leveraging antisense gene expression mitigates sources of extrinsic noise, allowing perfect adaptation (29). Abbreviations: iFFL, incoherent feedforward loop; iPSC, induced pluripotent stem cell; miRNA, microRNA; TF, transcription factor.

Random genomic integration methods support efficient integration of transgenic cargoes but introduce variability through copy number and loci of integrations. Using polytransfection, this variability can be exploited to survey optimal ratios of multi-vector systems (42). However, production of a cell product using polytransfection requires isolation of single clones with desired performance, which remains infeasible for many applications (49). Transcriptional activity varies across the nucleus in time even for identical genes (156). Local variation in regulatory proteins and nucleic acids may impact expression levels (157). For multi-vector circuits, distribution of synthetic circuit components across the genome may influence circuit performance by altering expression. For example, integrating co-regulated elements in spatially distinct regions of the nucleus may reduce their degree of co-regulation. Therefore, although random integration supports rapid transgenesis, these methods may not achieve robust performance for multi-vector systems without monoclonal selection (Figure 4a).

Alternatively, site-specific integration reduces transcriptional noise associated with copy number variation and integration locus by inserting transgenes at defined loci. Through selection of genomic loci resistant to epigenetic silencing, site-specific integration may also reduce transgene silencing (31). Nevertheless, the inefficiency of site-specific integration methods renders these methods infeasible for many applications that use primary cells. Additionally, the reliance on DNA DSBs to achieve sufficient levels of site-specific editing induces a large degree of genomic stress, which may compromise genomic integrity. To achieve robust dynamic behavior in mammalian cells, the field of synthetic biology must co-optimize both integration method and genetic cargo design to reduce variability associated with specific context effects, such as copy number and locus of integration (30, 158).

### 4.1. All-in-One Cargo Design

One solution to variability introduced through multi-vector systems is the design of all-in-one vectors that encode all transgenic components in a single payload (Figure 4a). All-in-one vectors enable a single integration step, performed randomly or at a predefined locus. All-in-one designs offer the advantage of controlling the relative copy numbers of genetic components (Figure 4a). Even for applications without complex genetic controllers, all-in-one vectors reduce the amounts of cytotoxic reagents required for cell engineering, reducing cell death and genotoxic stress. For example, reducing genetic cargo size of an AND-gate for identification of bladder cancer enabled delivery using an all-in-one AAV vector, improving delivery efficiency and circuit function (159). Although this research used a nonintegrating virus, we expect that similar increases in engineering efficiency would be observed with integrating viruses, such as retro- and lentiviruses.

For gene circuits sensitive to copy number variation, all-in-one systems make site-specific integration and selection more feasible. With the all-in-one recombinase-based gene expression cascade developed by Kim et al. (160), multiple copies of recombinase recognition sites on different chromosomes led to unpredictable outcomes, potentially related to translocations. Although reducing the amount of DNA or virus delivered in random integration grants some control over copy number, it reduces efficiency and does not ensure single copy integration. Instead, site-specific, all-in-one designs should facilitate more uniform and predictable responses, enabling more robust performance of circuits sensitive to nuclear concentrations and transgene copy number.

By improving the uniform performance of polyclonal lines, all-in-one cargo design may reduce the reliance on monoclonal cell lines for robust performance and improve the efficiency of integrating larger synthetic circuits. However, encoding many transcriptional units in close proximity can generate other emergent effects due to close-range transcriptional coupling. The unwinding of DNA during transcription generates regions of positive and negative supercoiling across genes that inhibit or enhance RNA polymerase binding, respectively. Multiple genes in close proximity can be biophysically coupled due to the accumulation of supercoiling (161–165). The effect of biophysical coupling is determined by factors including intergene spacing, gene length, and gene syntax (the relative order and orientation of genes) (161). Tuning these design characteristics can produce co-transcriptional positive or negative feedback, which may act to stabilize or destabilize all-in-one gene circuits (Figure 4b).

Specifically, the conventional tandem gene syntax, which orients genes in the same direction, is predicted to generate weak expression of the downstream gene when the upstream gene is active due to accumulation of positive supercoiling in the promoter region of the downstream gene (161). Accordingly, transcription of the upstream gene inhibits transcription of the downstream gene. Alternatively, the divergent syntax, which orients gene pairs facing each gene away from the other, supports high expression of both genes. Accumulation of negative supercoiling between divergent genes putatively acts as a form of positive feedback to amplify expression (161). Finally, the convergent syntax, which orients gene pairs facing each gene toward the other, is predicted to yield rapid either–or behavior via accumulation of positive supercoiling in the intergenic region (161). This may cause transcription of the two genes to be mutually inhibitory over a short timescale, although this expression variation may be dampened over longer timescales.

In co-local systems, cargoes can be designed to optimize syntax-specific dynamic behaviors. For example, the convergent architecture may be useful in designing an all-in-one toggle switch (Figure 4b). Previous studies suggested that a convergently oriented toggle switch exhibits increased negative feedback, sharper XOR logic, and increased separation between stable states (161, 162). Noise contributes to loss of toggle switch state stability (166), and the convergent syntax may stabilize attractor states to limit the frequency of noise-induced state switching by reducing both intrinsic and extrinsic noise (161). In comparison, the divergent architecture supports coordinated expression of two genes, which may improve inducible systems that rely on co-expression of a transcriptional activator (Figure 4b). Specifically, the Tet-On system uses the addition of doxycycline to trigger transcription from a Tet-responsive promoter via a constitutively expressed reverse tetracycline transactivator (rtTA) (167). Initial designs of an all-in-one Tet-On vector used tandem syntax, and expression of the rtTA downstream of the inducible expression unit lead to a poor signal-to-noise ratio (168, 169). Accumulation of positive supercoiling in the downstream promoter region may reduce expression of rtTA, impacting activation of the inducible gene. Changing the orientation from tandem to divergent eliminates this inhibitory interaction, resulting in strong expression of both the constitutive and inducible genes (169). Despite the improvements in inducible gene activation, the specific encoding of divergent syntax within a lentivirus resulted in lower viral titers, pointing to a trade-off in device performance and delivery efficiency. Alongside improvements to integration methods, supercoiling-informed design of all-in-one vectors could improve the speed and robustness of cell engineering by enabling single-step integration that promotes polyclonal performance.

### 4.2. Controllers for Circuit Robustness

For applications where all-in-one cargo design or site-specific integration is infeasible, genetic controllers can reduce variability introduced via copy number and locus of integration. Genetic controllers, such as the incoherent feedforward loop (iFFL) or the proportional-integral controller, mitigate disturbances from variation in the global or local transcription rate (Figure 4c). These controllers can be compactly added to genes of interest to buffer against expression differences due to transcriptional variation.

The iFFL possesses two circuit branches, an activation branch and a repression branch. In response to disturbances, both branches are simultaneously upregulated or downregulated, leading to disturbance rejection in the output. A common implementation of this controller is via synthetic microRNAs (miRNAs), which catalytically degrade or inhibit translation of mRNA transcripts containing cognate miRNA target sites (27, 170, 171). As transcription rate increases, more miRNA species are produced, degrading a fraction of the mRNA transcripts available for translation. Due to the small size of miRNAs and their target sites, miRNA-based controllers offer compact control. Although most implementations have been developed for transfection (27, 170, 171), a virally delivered miRNA-iFFL control circuit has been applied to limit the expression level of OCT4, a key transcription factor, during reprogramming of human fibroblasts to iPSCs (172). As designed, a miRNA-iFFL mitigates transcriptional noise. To control for both transcriptional and translational noise, endoRNase-based iFFL controllers can be used but require generally larger genetic cargoes (28). Combining the miRNA-based iFFL with additional negative feedback via transcriptional repression can further reduce expression heterogeneity across cell populations (173).

Integral control has also been used to buffer against disturbances and relies on negative regulation, like the iFFL. However, this mode of control must be implemented biologically via an annihilation reaction rather than a catalytic reaction such as miRNA-mediated inhibition (174). Instead, these controllers rely on the production of antisense RNA transcripts that bind with high affinity to sense mRNA transcripts, leading to degradation of both species (175). If antisense transcripts are generated in proportion to sense transcripts, the circuit exhibits perfect adaptation to transcriptional noise. Like the iFFL, pairing integral control with additional negative feedback to build a proportional-integral controller can further improve noise rejection (176), and this circuit design has been proposed for use in an insulin-secreting cell therapy (29).

These two control networks are specifically relevant for buffering transcriptional noise generated by genomic integration; however, we direct readers to Shakiba et al. (158) for a more complete discussion of biomolecular controllers for mitigating other context effects in mammalian cells. Through leveraging biophysical coupling and biomolecular controllers, designing genetic cargoes to mitigate sources of transcriptional noise will reduce expression variability across polyclonal cell populations for translation to cell therapies.

## 5. A VISION FOR FUTURE MAMMALIAN CELL ENGINEERING

The development of scalable culture technologies remains the largest bottleneck in translating cell-based therapies to the growing population of patients. For cell therapies, few tools exist for culturing, expanding, and processing large numbers of fragile cells. Cell culture automation is critical to effectively scaling production of clinically relevant cells from iPSCs and primary cells. However, stem cell culture techniques remain limited by the inherent fragility of the pluripotent stem cell state to spontaneous differentiation. Spontaneous differentiation substantially increases the cost of production through additional quality-control processes to identify products that fall out of specification. Although transgenes expand cellular capabilities, introducing transgenic cargoes further extends costly optimization and selection workflows for both allogeneic and autologous therapies. Thus, transgenesis should be designed to solve rather than compound the challenge of scalability.

Increasing transduction efficiency through vector design while limiting transgene expression will improve autologous therapies. Single vectors delivering multiple cargoes have recently established efficacy for CAR-T therapies in vitro and in mouse models (177). Online expression-level tuning via small molecules and other ligands may further improve the performance of autologous therapies. Tuning will support standardized vectors that can be tailored to match variation associated with donor cells. To reduce adverse effects from supraphysiological expression of transgenes, lower concentrations of viruses are currently used to achieve lower multiplicity of infection (MOI). Alternatively, genetic controllers that bound transgene expression can allow for higher MOI by mitigating overexpression-associated toxicity (Figure 4c). However, controlling transgene expression will not protect cells from the risks of insertional mutagenesis associated with high MOI. Thus, MOI and transduction efficiency remain a challenging trade-off in building online autologous therapies.

Offline construction of LP cell lines will expedite integration and provide rapid transgenesis in allogeneic stem cells, supporting efficient prototyping of synthetic systems in iPSCs and iPSC-derived cells. LP cell lines also facilitate standardized, high-throughput characterization of genetic cargoes. Combined with artificial intelligence and machine learning, high-throughput library screening will accelerate the design–build–test–learn cycle and the identification of optimal application-specific designs (44). Although current screening platforms can effectively identify the desired function for simple systems, such as induction of a single transgene, these platforms do not support effective characterization and identification of optimal dynamic circuit behaviors.

Dynamic systems such as homeostatic controllers and oscillators may enable the delivery of therapeutic molecules on defined schedules or in response to specific cues. However, establishing workflows to build and validate specific dynamics for monoclonal or polyclonal cells remains challenging. As we program more complex behaviors into cells, selection techniques must evolve beyond sorting based on steady-state ON/OFF expression levels. As observed in bet hedging in microbial populations (47, 178), temporally asynchronous and heterogeneous responses may not impede therapeutic function. Rather, variation may support robust function that exploits the inherent single-cell variability to stabilize the performance of the population. Microfluidic devices that support imaging for phenotyping over multiple cell cycles will be critical in evaluating how designs impact population dynamics and heterogeneity.

Translating stem cell–based therapies at scale requires processes that produce cells with defined identities, states, and functions. Application of synthetic biology design can make existing cell fate programming processes more efficient and precise to enable a new generation of cellular therapeutics. Differentiating cells to homogeneous populations with mature phenotypes remains difficult. With improvements in cell engineering, forward programming via transcription factor overexpression has been used to direct differentiation from iPSCs (179–181) and to control the generation of multiple cell types in organoids (182). Transcription factor–based control may improve reproducibility in iPSC-derived products by directly specifying activation of essential gene regulatory networks and reinforcing lineage commitment and maturation (183). Already, research efforts have pivoted toward the reproducibility of forward programming via transcription factor overexpression (184, 185). As more allogeneic pipelines and LP systems develop, iPSC-derived therapeutics will increasingly harness forward programming for robust and scalable cell fate determination (177).

Advances in synthetic biology, genomics, and automation will improve the precision and speed of cell engineering. Improved tools and increased understanding will equip researchers with unprecedented control of transgenesis and the integrated transgenes to generate a diverse array of therapeutic cells. By improving the predictability and performance of genetic designs, we can realize the potential of cell-based therapies at scale for diverse applications and patient populations.

## SUMMARY POINTS

1. Advances in synthetic biology, genomics, and automation are needed to translate transgenic systems to primary cells and stem cells at clinically relevant scales.
2. Techniques optimized for biologic production are not well-suited for cell-based therapies.
3. Offline design and engineering of allogeneic cells will improve the production speed and scalability of stem cell–based therapies.
4. Co-design of genetic cargo and transgenesis methods can improve engineering for a given cell type.
